# Supplementary material for: Explainable deep transfer learning model for disease risk prediction using high-dimensional genomic data
Source: PLoS Comput Biol. 2022 Jul 15;18(7):e1010328. doi: 10.1371/journal.pcbi.1010328 (PMC9328574; doi:10.1371/journal.pcbi.1010328)
Supplement: S1 Table — (PDF) [file pcbi.1010328.s001.pdf]

**Outcomes**	**Sample Size**	β_1	β_2	β_3	β_4
**Continuous**	$N = 1000$	$N(0, 2^2)$	$N(0, 8^2)$	0.5	10
	$N = 10000$	$N(0, (1/3)^2)$	$N(0, (4/3)^2)$	1/12	10
	$N = 100000$	$N(0, (1/80)^2)$	$N(0, (4/45)^2)$	1/150	1/30
**Binary**	$N = 1000$	$N(0, 2^2)$	$N(0, 8^2)$	0.5	10
	$N = 10000$	$N(0, (1/3)^2)$	$N(0, (4/3)^2)$	1/12	10
	$N = 100000$	$N(0, (3/250)^2)$	$N(0, (6/125)^2)$	0.01	0.02
