# Supplementary material for: Explainable deep transfer learning model for disease risk prediction using high-dimensional genomic data
Source: PLoS Comput Biol. 2022 Jul 15;18(7):e1010328. doi: 10.1371/journal.pcbi.1010328 (PMC9328574; doi:10.1371/journal.pcbi.1010328)
Supplement: S2 Table — (PDF) [file pcbi.1010328.s002.pdf]

Disease model	β_1	β_2	β_3	β_4
S_1: **Linear effects only**	$N(0, 0.5^2)$	$N(0, 2^2)$	0	0
S_2: **Non-linear effects only**	0	0	0.125	10
S_3: **Both linear and non-linear effects**	$N(0, 0.5^2)$	$N(0, 1)$	0.125	5
