# Supplementary material for: Explainable deep transfer learning model for disease risk prediction using high-dimensional genomic data
Source: PLoS Comput Biol. 2022 Jul 15;18(7):e1010328. doi: 10.1371/journal.pcbi.1010328 (PMC9328574; doi:10.1371/journal.pcbi.1010328)
Supplement: S3 Table — (PDF) [file pcbi.1010328.s003.pdf]

**Chromosome**	**Gene**	$P < 0.005$	$P < 0.001$	$P < 10^{-5}$
		DNN-screen		
5	PRRC1	0.850	0.850	0.100
19	APOC1	1.000	1.000	1.000
19	APOE	1.000	1.000	1.000
19	PVRL2	0.800	0.800	0.250
19	TOMM40	1.000	1.000	0.950
		SKAT-linear		
1	SPRR2G	1.000	0.650	0.000
2	LINC00471	1.000	0.800	0.000
7	DLD	1.000	0.700	0.000
8	ADAM28	1.000	1.000	0.000
10	PLAC9	0.950	0.650	0.000
11	APOC3	0.950	0.550	0.000
13	CPB2-AS1	1.000	0.950	0.000
13	CPB2	1.000	0.950	0.000
13	LINC01070	1.000	1.000	0.000
14	FBXO33	0.950	0.800	0.000
17	RPA1	1.000	0.800	0.000
19	APOC1	1.000	1.000	1.000
19	APOE	1.000	1.000	1.000
19	NTF4	1.000	0.950	0.100
19	SLC1A6	0.950	0.550	0.000
19	TOMM40	1.000	1.000	0.950
21	LOC101928269	1.000	0.850	0.000
		SKAT-optimal		
1	LINC00272	1.000	0.800	0.000
1	RHOC	0.850	0.800	0.000
1	SPRR2G	1.000	0.650	0.000
3	RNF7	1.000	0.550	0.000
5	NDUFS4	0.950	0.650	0.000
7	LINC01003	1.000	0.700	0.000
9	RANBP6	0.950	0.650	0.000
11	APOC3	1.000	0.850	0.000
11	KLHL35	1.000	0.650	0.000
11	LOC283299	1.000	0.700	0.000
11	OR52R1	0.800	0.650	0.000
16	C16orf82	0.800	0.550	0.000
17	FSCN2	1.000	0.550	0.000
19	ZNF473	0.850	0.550	0.000
22	BMS1P20	1.000	0.700	0.000
		ACAT		
1	PHTF1	0.650	0.550	0.000
1	RHOC	0.850	0.800	0.000
1	SPRR2G	1.000	0.650	0.000

Continued on next page

**Chromosome**	**Gene**	$P < 0.005$	$P < 0.001$	$P < 10^{-5}$
1	TGFB2	1.000	0.700	0.000
2	CFC1	0.700	0.650	0.000
2	CFC1B	0.700	0.650	0.000
3	GRIP2	0.950	0.800	0.000
5	PPP1R2P3	1.000	0.850	0.100
5	TIMD4	0.850	0.550	0.000
8	ADAM28	1.000	1.000	0.000
11	KCNQ1OT1	0.950	0.700	0.000
11	RAB30	1.000	0.850	0.100
13	LINC01070	1.000	1.000	0.000
14	FBXO33	0.950	0.550	0.000
16	C16orf82	0.700	0.550	0.000
16	EXOC3L1	0.950	0.650	0.000
17	ENDOV	0.950	0.850	0.000
17	RPA1	0.950	0.550	0.000
19	APOC1	1.000	1.000	1.000
19	APOE	1.000	1.000	1.000
19	NTF4	0.950	0.700	0.000
19	PVRL2	1.000	1.000	1.000
19	TOMM40	1.000	1.000	1.000
19	ZFP14	0.800	0.650	0.000
19	ZNF146	0.850	0.700	0.000
19	ZNF473	1.000	0.850	0.000
19	ZNF565	1.000	0.950	0.500
21	LOC101928269	1.000	0.950	0.000
