# Supplementary material for: Explainable deep transfer learning model for disease risk prediction using high-dimensional genomic data
Source: PLoS Comput Biol. 2022 Jul 15;18(7):e1010328. doi: 10.1371/journal.pcbi.1010328 (PMC9328574; doi:10.1371/journal.pcbi.1010328)
Supplement: S4 Table — (PDF) [file pcbi.1010328.s004.pdf]

Chromosome	Gene	$P < 0.005$	$P < 0.001$	$P < 10^{-5}$
		DNN-screen		
2	ACKR3	0.650	0.650	0.000
5	ADAMTS19	0.650	0.650	0.000
11	TSGA10IP	0.650	0.650	0.000
17	NPLOC4	0.650	0.650	0.000
19	APOC1	1.000	1.000	1.000
19	APOE	1.000	1.000	1.000
19	PVRL2	0.850	0.850	0.250
19	TOMM40	0.850	0.850	0.850
21	DIP2A	0.800	0.800	0.200
		SKAT-linear		
1	MIR4781	1.000	0.700	0.000
1	NTNG1	1.000	0.800	0.000
2	BCL11A	1.000	0.800	0.000
2	NXPH2	0.950	0.650	0.000
4	DKFZP434I0714	0.950	0.550	0.000
5	ADAMTS19	1.000	0.850	0.000
6	SLC2A12	1.000	0.950	0.000
7	TOMM7	1.000	0.800	0.000
8	EBAG9	1.000	0.700	0.000
9	OMD	0.850	0.550	0.000
12	CDK2AP1	0.950	0.550	0.000
16	GLG1	1.000	0.800	0.000
17	ASB16-AS1	1.000	0.950	0.000
17	ASB16	1.000	1.000	0.000
17	ATXN7L3	1.000	1.000	0.000
17	C17orf53	1.000	1.000	0.000
17	TMUB2	1.000	1.000	0.000
19	APOC1	1.000	1.000	1.000
19	APOE	1.000	1.000	1.000
19	GAMT	1.000	0.950	0.000
19	LRP3	1.000	1.000	0.000
19	TOMM40	1.000	1.000	0.650
19	ZNF805	1.000	1.000	0.000
21	TRAPPC10	1.000	0.700	0.000
		SKAT-optimal		
1	B3GALNT2	0.800	0.650	0.000
1	FMO2	1.000	0.850	0.000
1	LDLRAD1	1.000	0.800	0.100
1	MIR4781	1.000	0.700	0.000
1	TCEANC2	1.000	0.950	0.100
2	LINC00486	1.000	0.950	0.200
2	LOC100271832	0.950	0.700	0.000
2	LOC102724058	1.000	0.800	0.000

Continued on next page

Chromosome	Gene	$P < 0.005$	$P < 0.001$	$P < 10^{-5}$
5	LOC101927134	1.000	0.700	0.000
5	LOC101929380	1.000	0.800	0.000
5	SPINK6	1.000	0.700	0.000
6	C6orf57	0.950	0.700	0.000
6	FAM135A	1.000	1.000	0.000
6	GMNN	1.000	0.700	0.000
8	FAM83A	0.950	0.800	0.000
8	SOX7	1.000	0.850	0.000
8	TRHR	0.950	0.850	0.000
8	ZHX1-C8orf76	1.000	0.800	0.000
9	B4GALT1-AS1	1.000	0.550	0.000
9	SH3GL2	0.950	0.700	0.000
11	CCND1	1.000	0.950	0.000
11	GPR83	1.000	0.650	0.000
11	IFT46	0.850	0.550	0.000
11	ORAOV1	1.000	0.850	0.000
12	APOF	0.950	0.650	0.000
12	ARL6IP4	1.000	0.650	0.000
12	C1RL-AS1	1.000	0.800	0.000
12	FLJ41278	0.950	0.550	0.000
12	LINC01252	0.850	0.550	0.000
12	PITPNM2	0.950	0.800	0.000
12	SETD8	0.850	0.550	0.000
13	ESD	0.850	0.850	0.000
13	PDX1-AS1	1.000	0.700	0.000
15	DCAF13P3	1.000	0.550	0.000
16	IL34	1.000	0.700	0.000
17	AATK-AS1	0.950	0.650	0.000
17	CD7	0.950	0.800	0.000
17	HDAC5	1.000	0.850	0.000
17	LOC101927728	1.000	0.700	0.000
18	KCNG2	1.000	0.950	0.000
19	MIDN	1.000	1.000	0.000
19	YJEFN3	1.000	0.950	0.200
20	CST11	1.000	0.700	0.000
		ACAT		
1	PHTF1	0.650	0.550	0.000
1	RHOC	0.850	0.800	0.000
1	SPRR2G	1.000	0.650	0.000
1	TGFB2	1.000	0.700	0.000
2	CFC1	0.700	0.650	0.000
2	CFC1B	0.700	0.650	0.000
3	GRIP2	0.950	0.800	0.000
5	PPP1R2P3	1.000	0.850	0.100
5	TIMD4	0.850	0.550	0.000
8	ADAM28	1.000	1.000	0.000

Continued on next page

**Chromosome**	**Gene**	$P < 0.005$	$P < 0.001$	$P < 10^{-5}$
11	KCNQ1OT1	0.950	0.700	0.000
11	RAB30	1.000	0.850	0.100
13	LINC01070	1.000	1.000	0.000
14	FBXO33	0.950	0.550	0.000
16	C16orf82	0.700	0.550	0.000
16	EXOC3L1	0.950	0.650	0.000
17	ENDOV	0.950	0.850	0.000
17	RPA1	0.950	0.550	0.000
19	APOC1	1.000	1.000	1.000
19	APOE	1.000	1.000	1.000
19	NTF4	0.950	0.700	0.000
19	PVRL2	1.000	1.000	1.000
19	TOMM40	1.000	1.000	1.000
19	ZFP14	0.800	0.650	0.000
19	ZNF146	0.850	0.700	0.000
19	ZNF473	1.000	0.850	0.000
19	ZNF565	1.000	0.950	0.500
21	LOC101928269	1.000	0.950	0.000
