# Supplementary figures and images for: Explainable deep transfer learning model for disease risk prediction using high-dimensional genomic data
Source: PLoS Comput Biol. 2022 Jul 15;18(7):e1010328. doi: 10.1371/journal.pcbi.1010328 (PMC9328574; doi:10.1371/journal.pcbi.1010328)

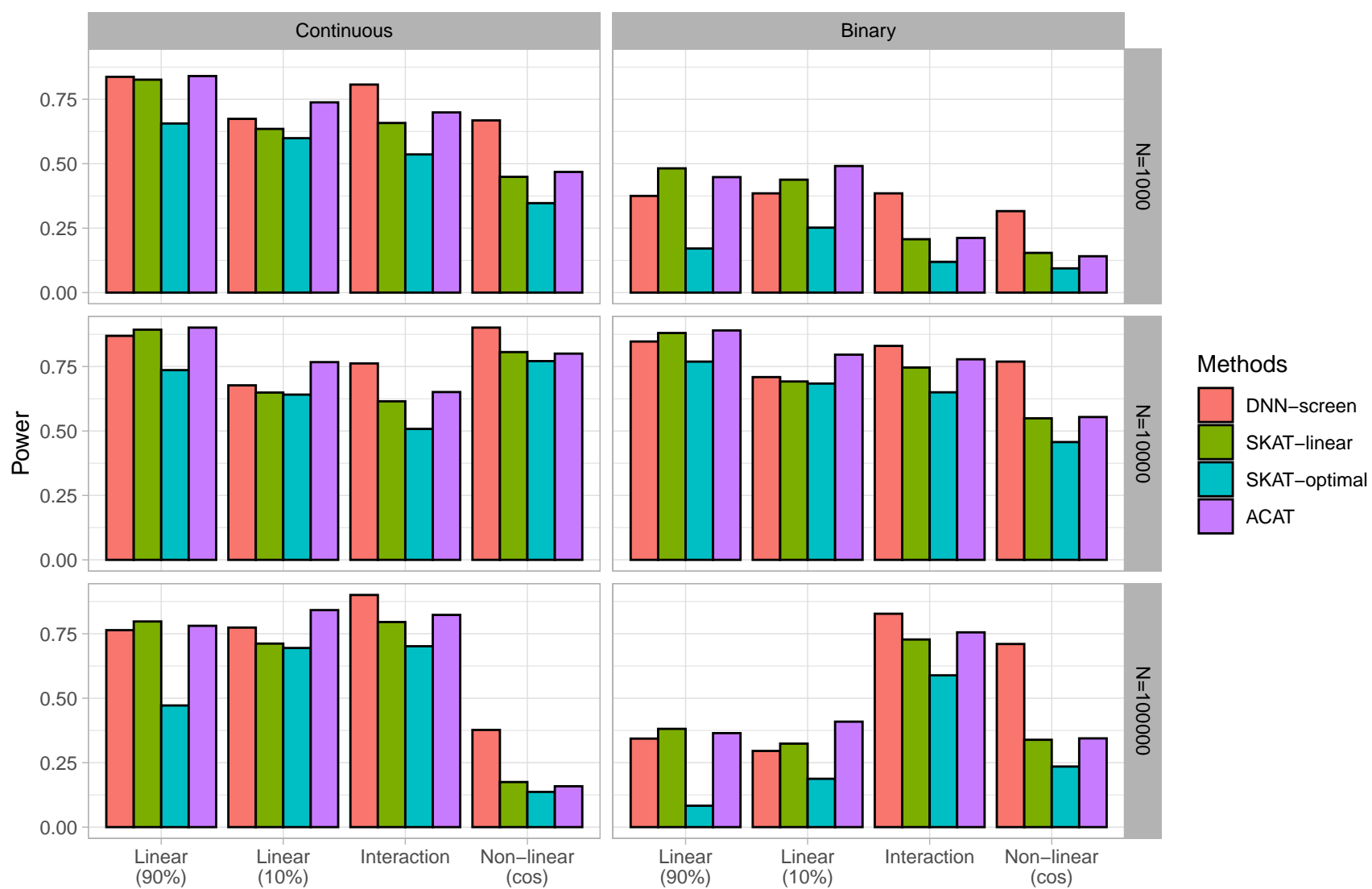

Supplement: S1 Fig — (PDF) [file pcbi.1010328.s005.pdf]

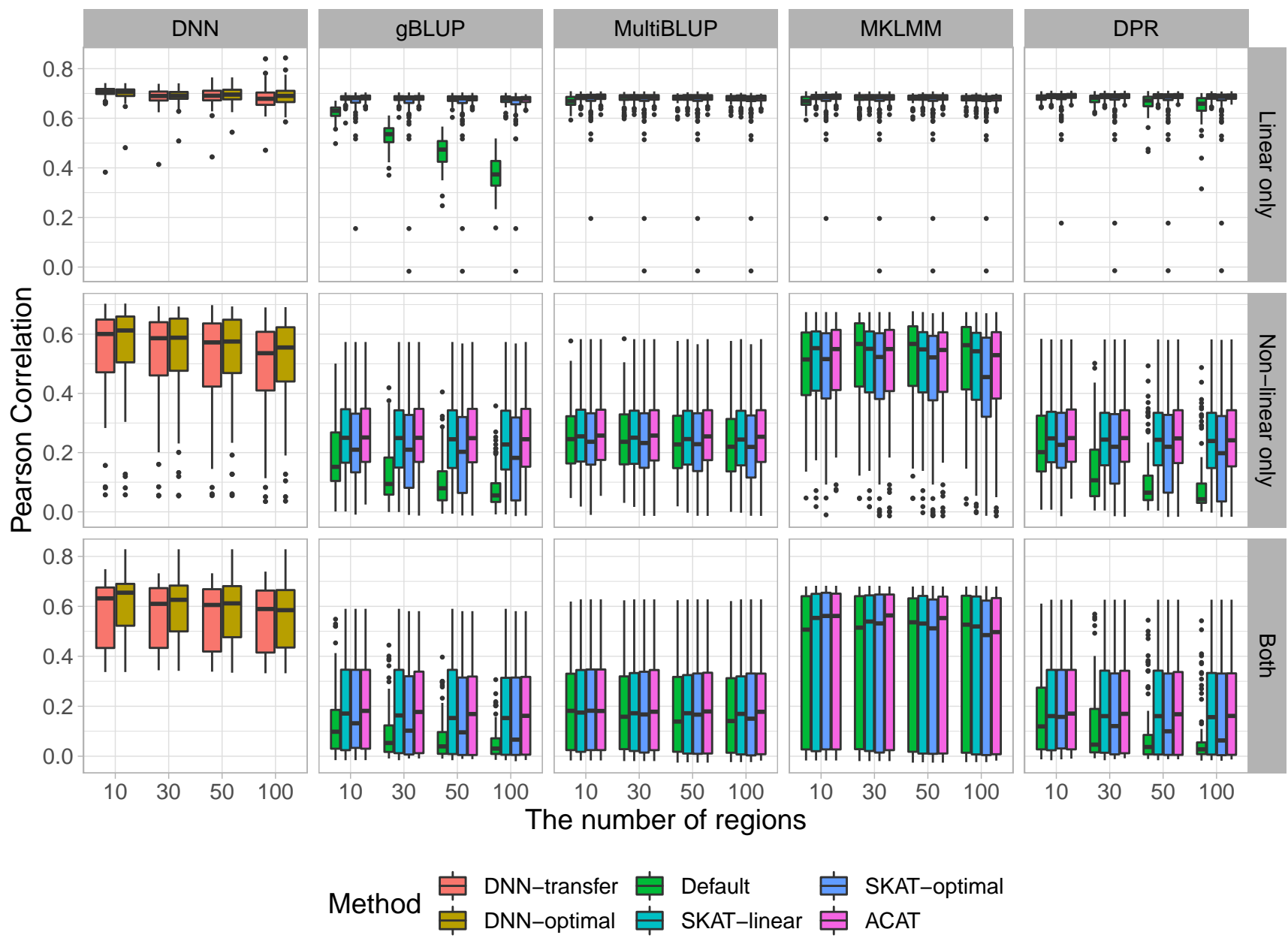

Supplement: S2 Fig — Genes with p-values less than 0.005 are considered significant. (PDF) [file pcbi.1010328.s006.pdf]

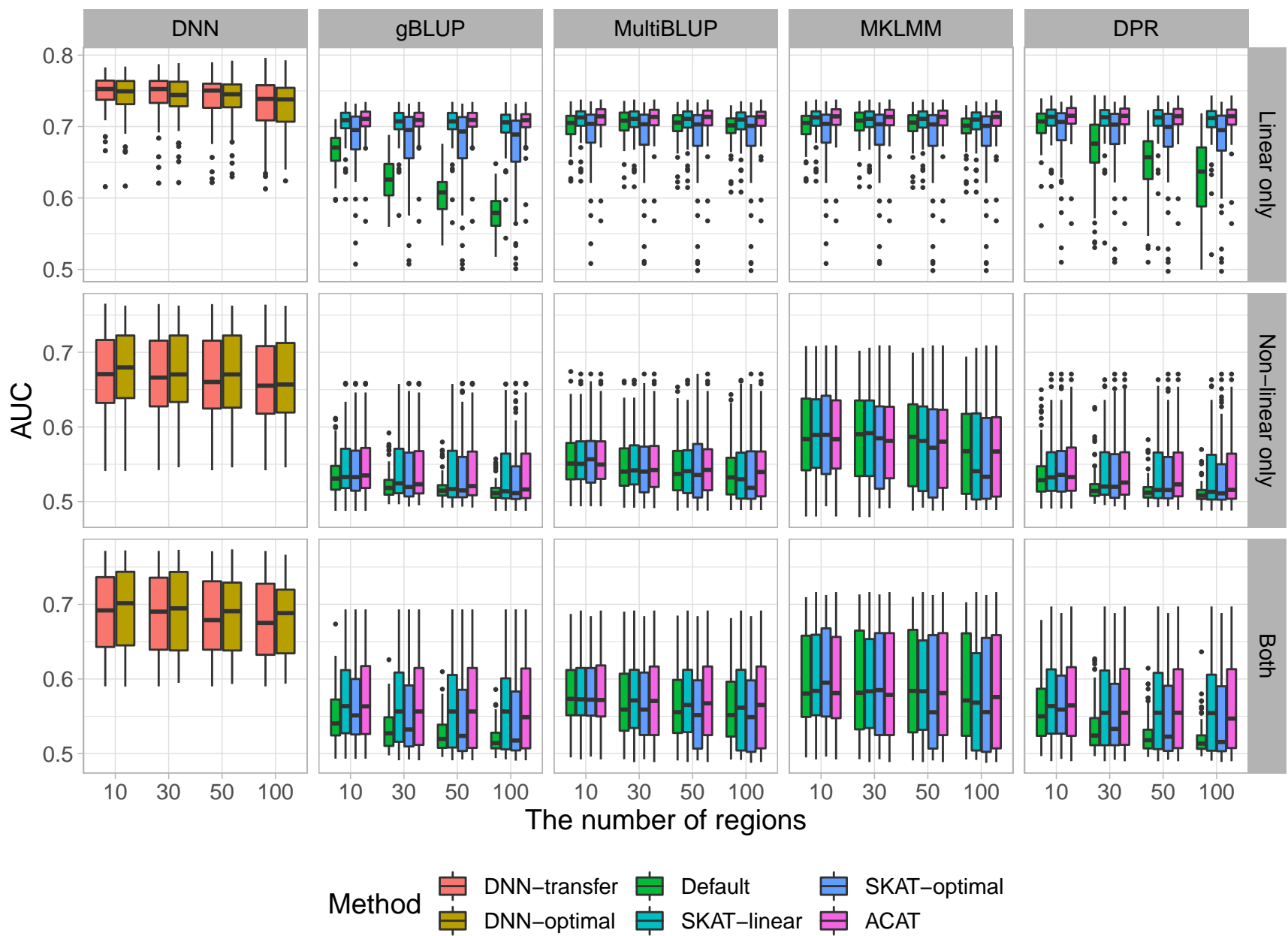

Supplement: S3 Fig — Genes with p-values less than 0.005 are considered significant. (PDF) [file pcbi.1010328.s007.pdf]

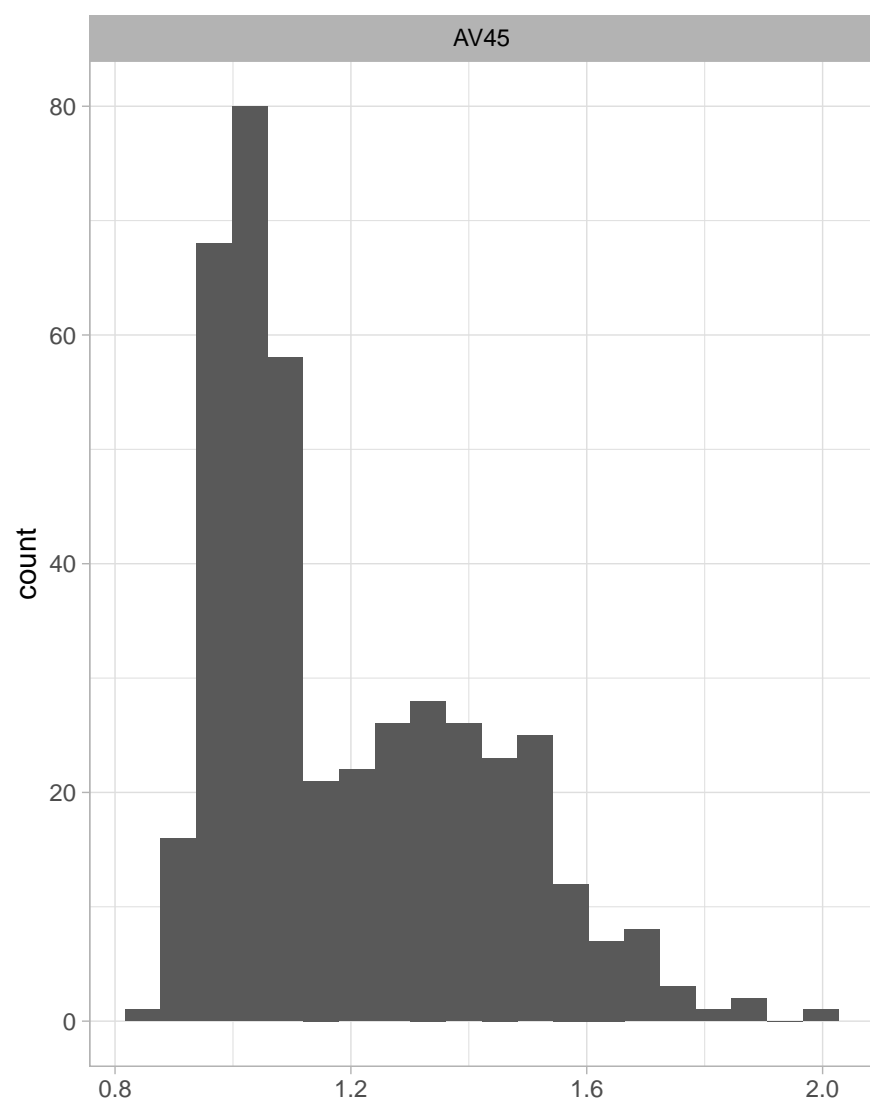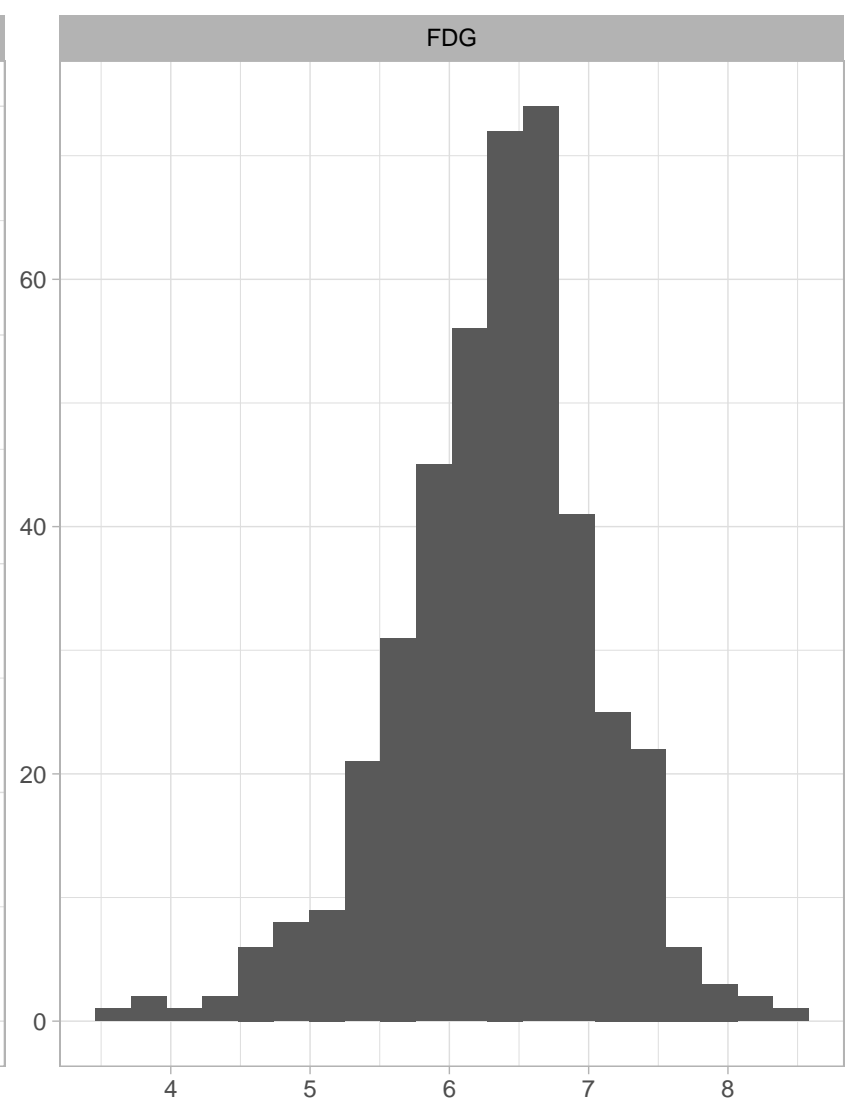

Supplement: S4 Fig — (PDF) [file pcbi.1010328.s008.pdf]

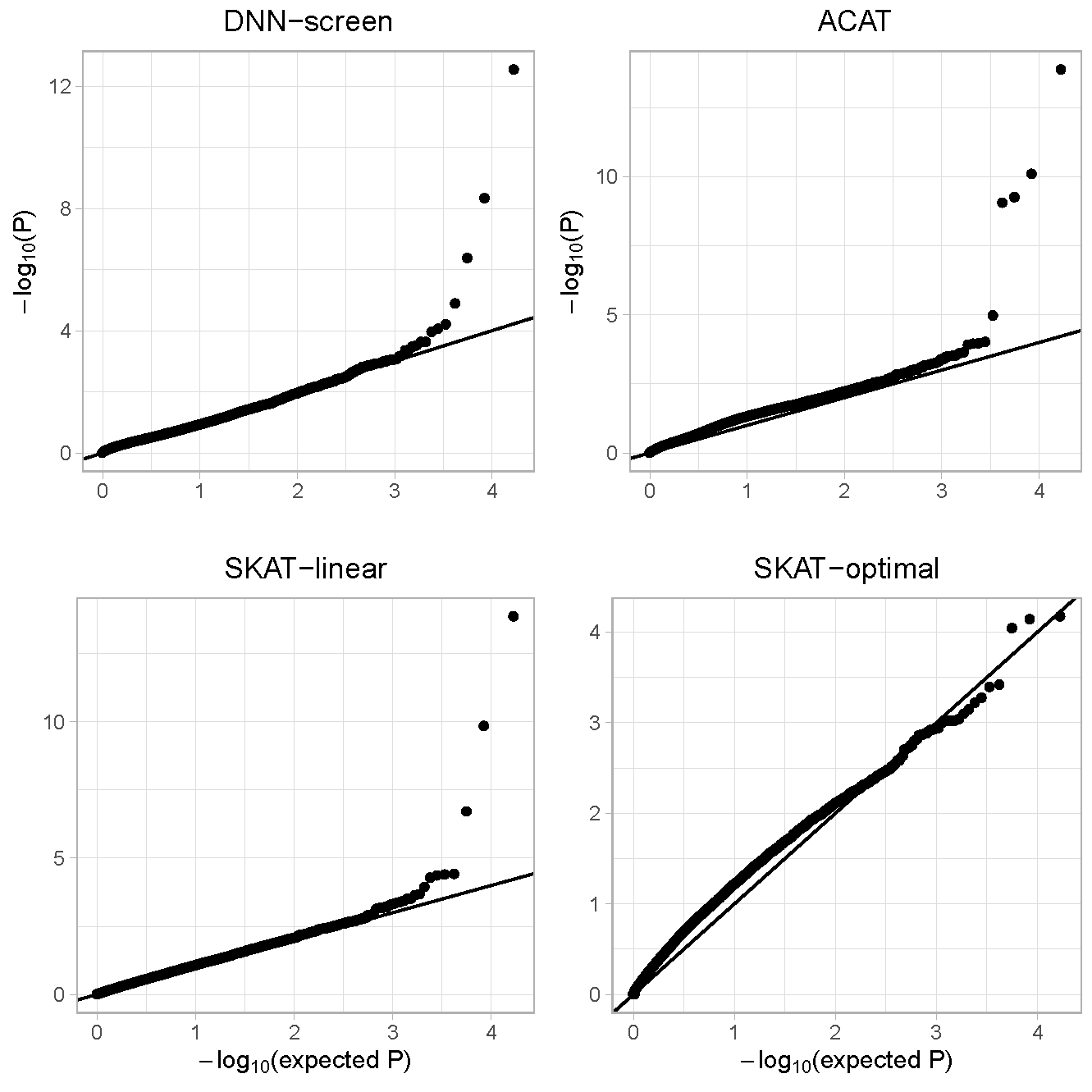

Supplement: S5 Fig — (PNG) [file pcbi.1010328.s009.png]

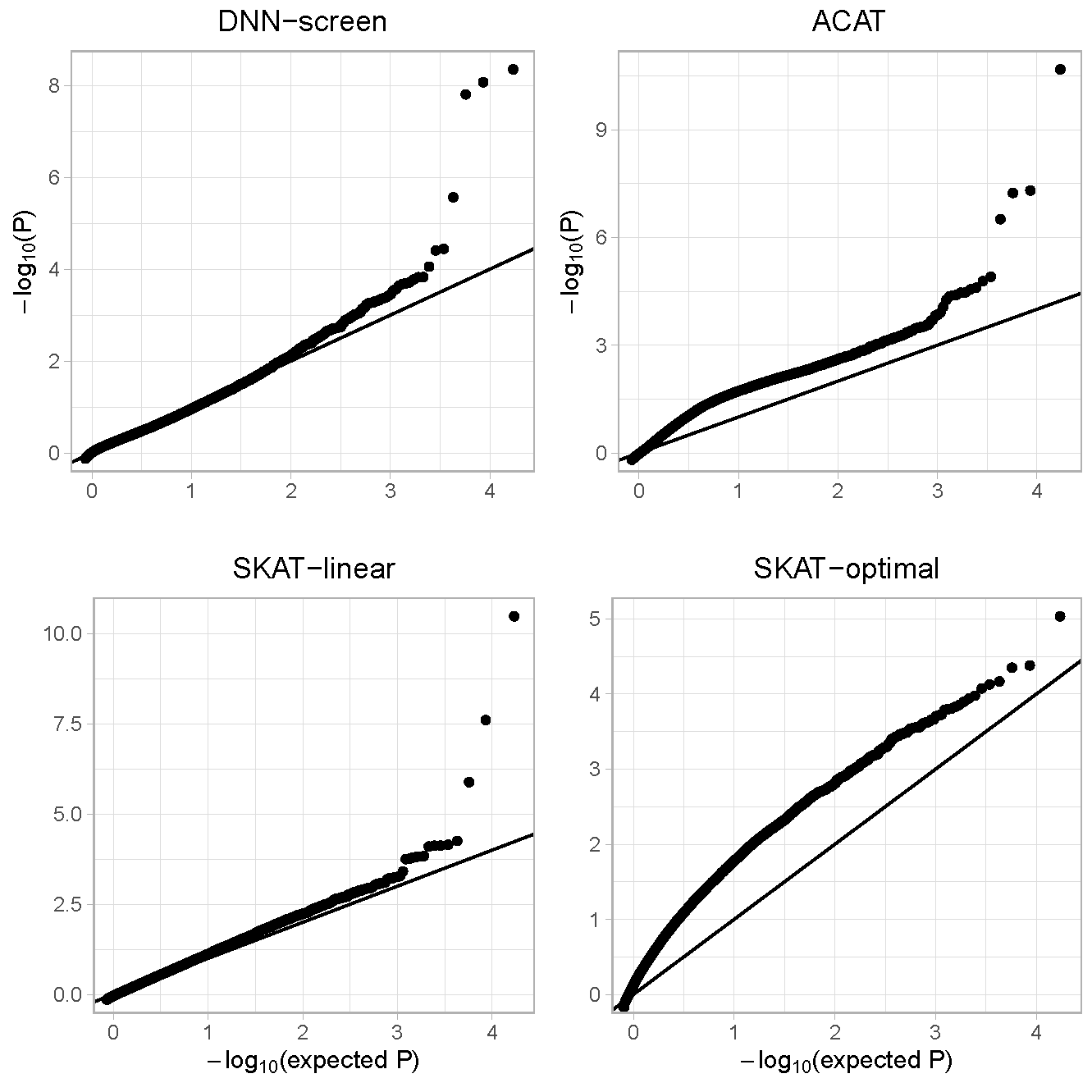

Supplement: S6 Fig — (PNG) [file pcbi.1010328.s010.png]

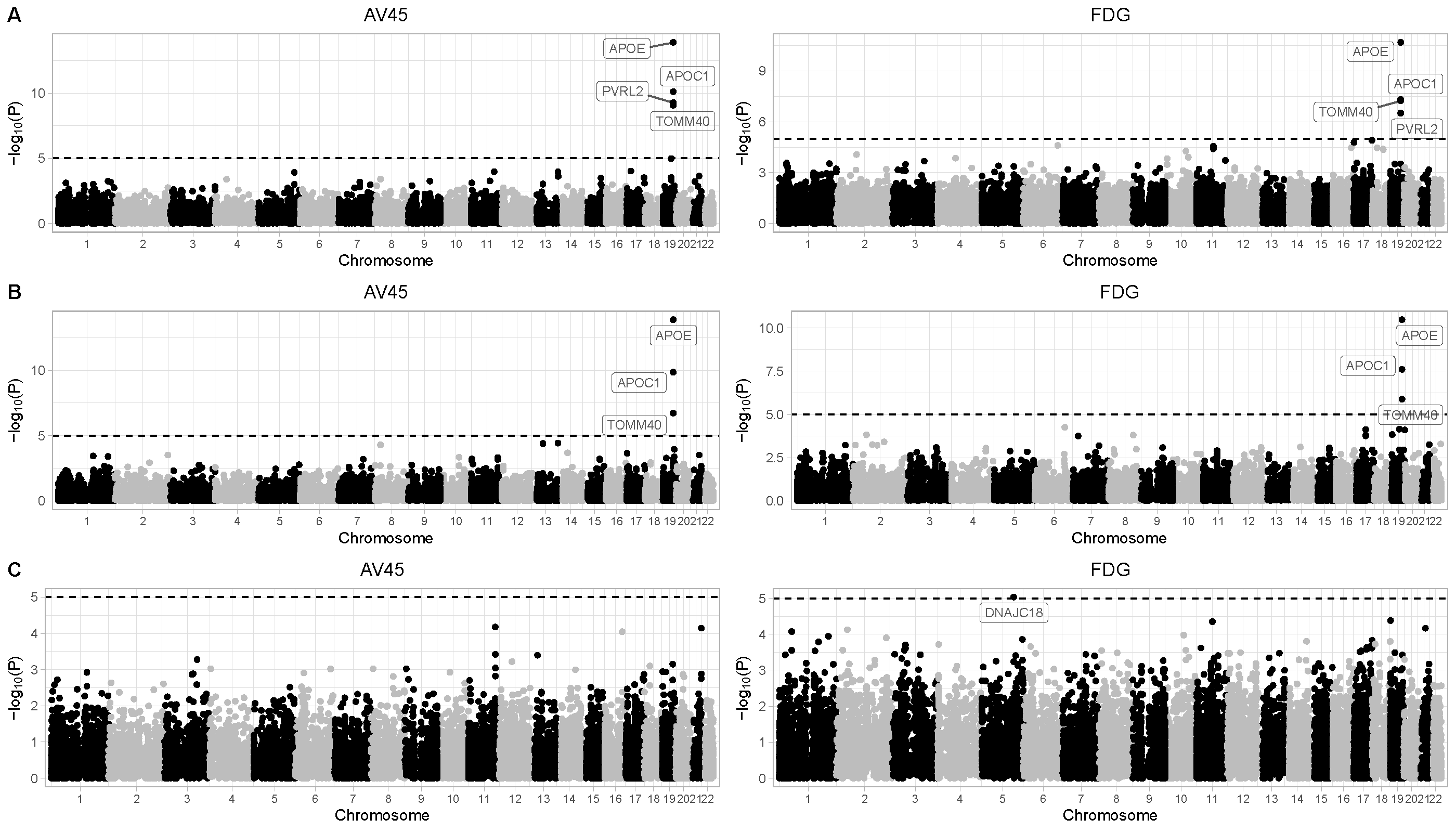

Supplement: S7 Fig — (PNG) [file pcbi.1010328.s011.png]

Pearson Correlation

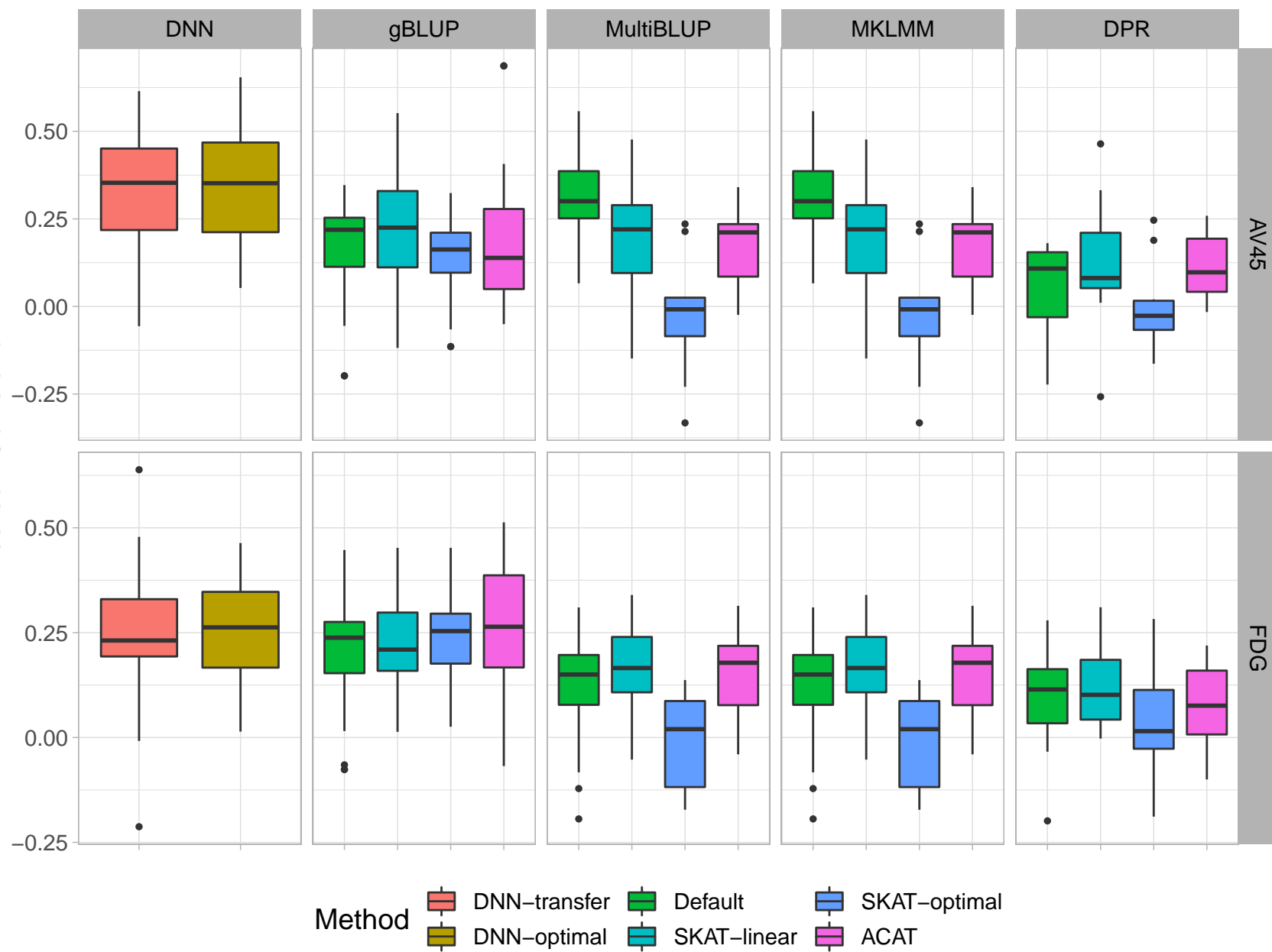

Supplement: S8 Fig — Genes are pre-selected under the p-value threshold of 0.005 for DNN-transfer, SKAT-linear, SKAT-optimal and ACAT. (PDF) [file pcbi.1010328.s012.pdf]

**A**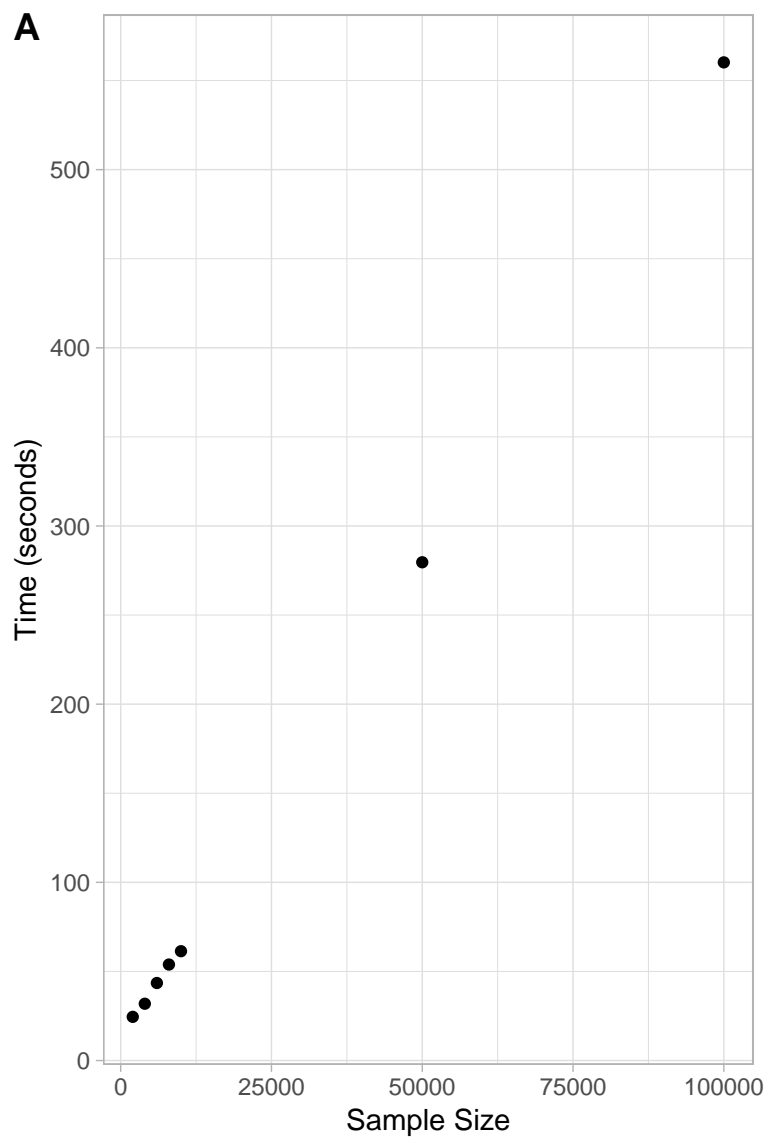**B**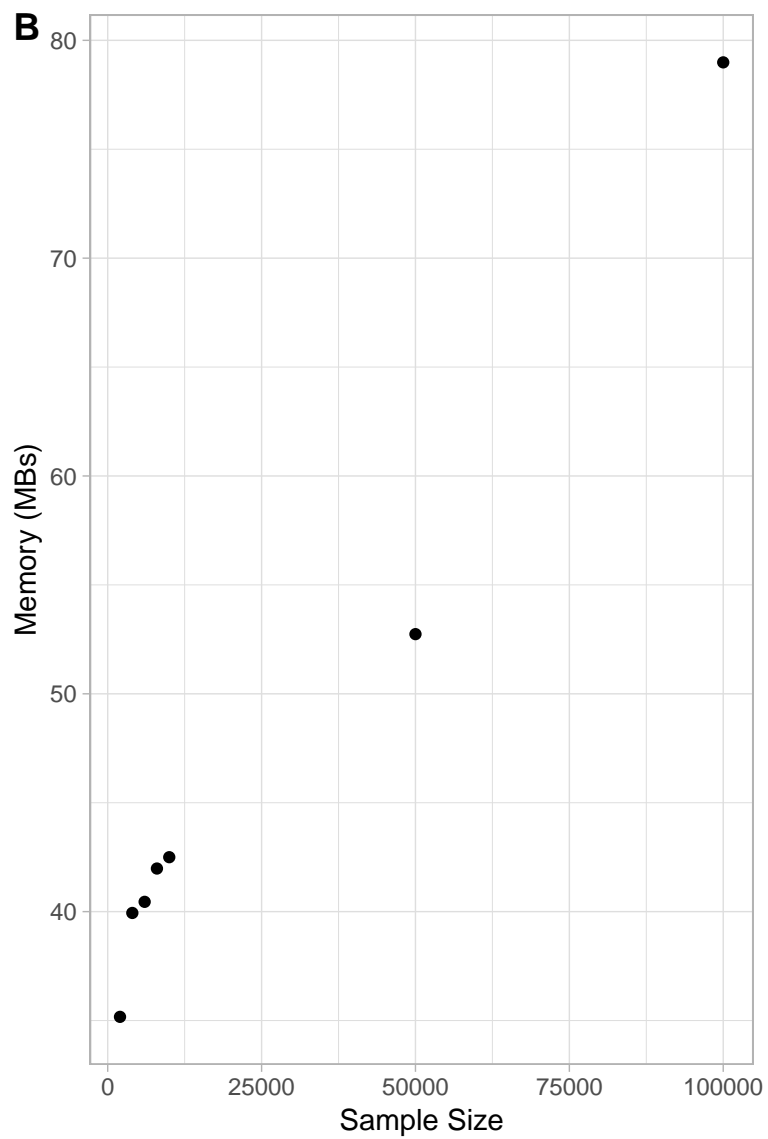

Supplement: S9 Fig — (PDF) [file pcbi.1010328.s013.pdf]
